# Supplementary material for: Characteristics of pediatric emergency department frequent visitors and their risk of a return visit: A large observational study using electronic health record data
Source: PLoS One. 2022 Jan 27;17(1):e0262432. doi: 10.1371/journal.pone.0262432 (PMC8794145; doi:10.1371/journal.pone.0262432)
Supplement: S1 Table — * Haemodynamic support (e.g. significant IV fluid in case of hypotension, blood administration or control of major bleeding) or emergency medications (e.g. atropine, adenosine, inotropics, epinephtrine, nalaxon). (PDF) [file pone.0262432.s002.pdf]

S1 Table. True severity classification [18]

Severity category	Items	Time before being seen by a physician
High severity	- Mortality at the ED, and/or<br>- ICU admission immediately after the ED visit, and/or<br>- Immediate lifesaving interventions*	< 10 minutes
Intermediate severity	- Hospital admission immediately after the ED visit, and/or<br>- IV medication or fluids or inhalation medication at the ED, and/or<br>- > 1 of the following: radiology, lab test, oral medication	< 60 minutes
Low severity	- None of the above	= > 60 minutes

** Haemodynamic support (e.g. significant IV fluid in case of hypotension, blood administration or control of major bleeding) or emergency medications (e.g. atropine, adenosine, inotropics, epinephtrine, nalaxon)*
