# Supplementary material for: Characteristics of pediatric emergency department frequent visitors and their risk of a return visit: A large observational study using electronic health record data
Source: PLoS One. 2022 Jan 27;17(1):e0262432. doi: 10.1371/journal.pone.0262432 (PMC8794145; doi:10.1371/journal.pone.0262432)
Supplement: S2 Table — NA not applicable, C-CD Complex Chronic Disease, NC-CD Non-Complex Chronic Disease, CD Chronic Disease. *The examples used in this document to illustrate definitions of medical complexity are intended to demonstrate characteristics specified in the definition/descriptions. It is not our intention to imply that specific diseases and conditions are by default linked to the categories that they were used to illustrate. (PDF) [file pone.0262432.s003.pdf]

S2 Table. Pediatric Medical Complexity Algorithm [21]

COE4CCN Consensus Definitions of 3 Levels of Medical Complexity

	Condition Description	Examples *
**Children with C-CD**		
	**Significant chronic conditions in two or more body systems:** **Significant chronic condition** is defined as a physical, mental or developmental condition that can be expected to last at least a year, will use health care resources above the level for a healthy child, require treatment for control of the condition, and the condition can be expected to be episodically or continuously debilitating.<br>**Body systems** include cardiac, craniofacial, dermatologic, endocrinologic, gastrointestinal, genetic, genitourinary, hematologic, immunologic, mental health, metabolic, musculoskeletal, neurologic, ophthalmologic, otologic, pulmonary/respiratory, and renal.	type 1 diabetes and static encephalopathy; type 1 diabetes and depression; developmental delay and chronic pulmonary conditions
OR	A progressive condition that is associated with deteriorating health with a decreased life expectancy in adulthood.	muscular dystrophy, cystic fibrosis, paraplegia, quadriplegia, malignancy
OR	Continuous dependence on technology for at least six months.	tracheostomy +/- ventilator assistance, renal dialysis, gastrostomy tube, CSF shunt
OR	Malignancies: Progressive or metastatic malignancies that impact life function. Exclude those in remission for more than 5 years.	lymphoma, leukemia, brain tumor
**Children with NC-CD**		
	Chronic Conditions that last at least one year: These conditions are commonly lifelong but can be episodic with periods of good health in between episodes. They include physical, developmental, or mental health conditions that may persist into adulthood but may also resolve either secondary to the natural history of the disease or as a result of surgical intervention. These conditions involve a single body system, are not progressive, can vary widely in severity and result in highly variable health care utilization.	type 1 diabetes, atrial septal defect, asthma, depression, ADHD
**Children without CD**		
	Acute Non-Chronic Conditions: A physical, developmental or mental health condition that is not expected to last more than a year. These children may temporarily (for < 1 year) utilize health care resources above the normal level for a healthy child.	ear infection, pneumonia, diarrhea and dehydration, bronchiolitis
	Healthy: No acute or chronic health conditions. These children do not utilize health care resources above the normal level for a healthy child.	N/A

NA not applicable, C-CD Complex Chronic Disease, NC-CD Non-Complex Chronic Disease, CD Chronic Disease

*The examples used in this document to illustrate definitions of medical complexity are intended to demonstrate characteristics specified in the definition/descriptions. It is not our intention to imply that specific diseases and conditions are by default linked to the categories that they were used to illustrate.
