# Supplementary material for: Characteristics of pediatric emergency department frequent visitors and their risk of a return visit: A large observational study using electronic health record data
Source: PLoS One. 2022 Jan 27;17(1):e0262432. doi: 10.1371/journal.pone.0262432 (PMC8794145; doi:10.1371/journal.pone.0262432)
Supplement: S3 Table — C Communicable NC Non-Communicable. (PDF) [file pone.0262432.s004.pdf]

S3 Table. Division of acute diagnosis

Main category	Subgroup	Acute diagnosis group
Infectious	C- ear and throat	Otitis media
		Tonsillitis
		Other upper respiratory infections
	C- respiratory	Pneumonia
		Other lower respiratory infections
	C- eye	Eye infections
	C- urinary	Urinary tract infections
		Genital infections
		Other urogenital infections
	C- gastrointestinal	Appendicitis
		Other gastrointestinal infections
	C- skin	Childhood diseases
		Other skin infections
	C- other infections	Bone infections
		Fever of unknown origin
		Meningitis
		Seizure
		Sepsis
		Other infections
Intoxication/Injury	NC- intoxication/Injury	Accidental intoxications
		Assaults
		Burns and scalds
		Foreign body
		Injuries
		Major traumas
		Minor traumas
		Radius traumas
		Other substance intoxication
		Other external cause
Other	NC- circulator	Collapse
		Other circulator problems
	NC- congenital malformation	Problems due to congenital malformation
	NC- endocrinological	Diabetic
		Other endocrinological problems
	NC- eye/ear	Ear problems
		Eye problems
	NC- gastrointestinal	Herniation (except from hernia diaphragmatic)
		Volvulus
		Bowel function disorder
		Inflammatory Bowel diseases
		Other gastrointestinal problems
	NC- hematological	Sickle crisis
		Other hematological problems
	NC- muscle or joint	Muscle or joint problems

	NC- neoplasms	Problems due to neoplasms
	NC- neurological	Insult<br>Headache<br>Other neurological problems
	NC- perinatal problems	Perinatal problems
	NC- psychological	Mental health problems
	NC- respiratory	Asthma<br>Other respiratory problems
	NC- skin	Eczema<br>Other skin problems
	NC- urogenital	Testicular torsion<br>Kidney problems<br>Other urogenital problems

C Communicable NC Non-Communicable
