# Supplementary material for: Characteristics of pediatric emergency department frequent visitors and their risk of a return visit: A large observational study using electronic health record data
Source: PLoS One. 2022 Jan 27;17(1):e0262432. doi: 10.1371/journal.pone.0262432 (PMC8794145; doi:10.1371/journal.pone.0262432)
Supplement: S4 Table — C Communicable NC Non-Communicable. (PDF) [file pone.0262432.s005.pdf]

S4 Table. Diagnose corresponding to comorbidity

Comorbidity	Diagnosis
Cardiac	NC - Circulator
Craniofacial	NC – Congenital malformation
Dermatological	NC – Skin<br>C - Skin
Gastrointestinal	C – gastrointestinal<br>NC – gastrointestinal
Genetic	NC – Congenital malformation
Hematological	NC – hematological
Endocrinological	NC – endo
Genito	C – Urinary<br>NC – Urogenital
Immunological	All communicable
Malignity	NC – neoplasms
MH	NC – psychological
Neuro	NC – neurological
Muscolo	NC – Muscle or Joint
Metab	NC – Congenital malformation
Opthal	C – eye<br>NC – eye/ear
Otol	C – ENT<br>NC – eye/ear
Pulresp	C – Resp<br>NC – Resp
Renal	C – Urinary<br>NC - Urogenital
