# Supplementary material for: Characteristics of pediatric emergency department frequent visitors and their risk of a return visit: A large observational study using electronic health record data
Source: PLoS One. 2022 Jan 27;17(1):e0262432. doi: 10.1371/journal.pone.0262432 (PMC8794145; doi:10.1371/journal.pone.0262432)
Supplement: S5 Table — *Because children could have more than one diagnoses category rows do not add up to 100%. Diagnoses corresponding to the body system of the comorbidity are marked bold. NC: Non-Communicable; I: Intoxication/injuries; C: Communicable. (PDF) [file pone.0262432.s006.pdf]

S5 Table. Reason for visiting the ED split by comorbidity at patient level

Table Reason for visiting the ED split by comorbidity at patient level

Comorbidities (number of children)	Reason for visit*			Reason for visiting the ED		
	NC	I	C	Most frequent	2nd most frequent	3th most frequent
**Neurologic (830)**	90.4%	12.5%	27.8%	**NC-Neurological (46.5%)**	NC-Unspecified diagnosis (19.3%)	C-Unspecified infection (14.9%)
- **FVs (95)**	72.7%	6.6%	40.4%	**NC-Neurological (27.5%)**	C-Unspecified infection (19.8%)	NC-Unspecified diagnosis (19.1%)
**Cardiac (346)**	73.7%	8.1%	34.0%	**NC-Circulation (27.0%)**	C-Unspecified infection (17.8%)	NC-Unspecified diagnosis (12.7%)
- **FVs (31)**	71.5%	6.7%	40.9%	**NC-Circulation (19.0%)**	C-Unspecified infection (18.3%)	NC-Unspecified diagnosis (15.3%)
**Pulmonal (327)**	76.0%	5.9%	34.6%	**NC-Respirator (43.5%)**	C-Unspecified infection (13.3%)	NC-Unspecified diagnosis (12.7%)
- **FVs (41)**	80.8%	1.9%	38.6%	**NC-Respirator (42.4%)**	NC-Unspecified diagnosis (18.6%)	**C-Respirator (15.6%)**
**Musculo/skeletal (282)**	61.5%	21.8%	30.4%	NC-Intoxication/Injury (21.8%)	C-Unspecified infection (12.1%)	NC-Unspecified diagnosis (11.8%)
- **FVs (42)**	74.0%	8.9%	36.3%	NC-Unspecified diagnosis (18.0%)	NC-Respirator (15.4%)	C-Unspecified infection (14.5%)
**Otologic (272)**	62.9%	14.5%	43.7%	C-Unspecified infection (17.9%)	**NC-Ear/Eye (14.8%)**	NC-Unspecified diagnosis (14.6%)
- **FVs (34)**	71.0%	3.4%	51.0%	C-Unspecified infection (23.9%)	NC-Unspecified diagnosis (19.7%)	NC-Neurological (14.2%)
**Hematologic (269)**	61.7%	34.2%	18.7%	**NC-Hematological (34.4%)**	NC-Intoxication/Injury (34.2%)	C-Unspecified infection (12.9%)
- **FVs (39)**	54.9%	34.7%	25.4%	NC-Intoxication/Injury (34.7%)	**NC-Hematological (24.2%)**	C-Unspecified infection (16.3%)
**Gastrointestinal (271)**	73.4%	5.8%	33.6%	**NC-Gastrointestinal (40.7%)**	**C-Gastrointestinal infection (15.0%)**	NC-Unspecified diagnosis (11.4%)
- **FVs (39)**	68.8%	1.9%	43.4%	**NC-Gastrointestinal (32.2%)**	**C-Gastrointestinal infection (21.5%)**	NC-Unspecified diagnosis (16.0%)
**Malignancy (203)**	58.9%	10.2%	44.4%	C-Unspecified infection (28.4%)	**NC-Neoplasm (25.1%)**	NC-Intoxication/Injury (10.3%)
- **FV (45(22.2%))**	56.4%	6.3%	51.4%	C-Unspecified infection (37.2)	**NC-Neoplasm (27.5%)**	NC-Unspecified diagnosis (9.4%)
**Renal (188)**	65.1%	6.0%	46.0%	**NC-Urogenital (21.1%)**	NC-Unspecified diagnosis (17.2%)	**C-Urinary infection (16.4%)**
- **FVs (40)**	52.2%	3.9%	58.9%	C-Unspecified infection (23.6%)	C-Gastrointestinal infection (16.7%)	NC-Unspecified diagnosis (15.7%)
**Genetic (168)**	67.3%	9.1%	41.3%	C-Unspecified infection (16.6%)	NC-Unspecified diagnosis (15.1%)	C-Gastrointestinal infection (12.2%)
- **FVs (26)**	63.4%	2.1%	55.1%	NC-Unspecified diagnosis (23.3%)	C-Unspecified infection (20.6%)	C-Gastrointestinal infection (19.9%)
**Ophthalmologic (168)**	70.8%	19.9%	42.9%	NC-Unspecified diagnosis (23.0%)	**NC-Ear/Eye (19.8%)**	C-Unspecified infection (14.9%)
- **FVs (27)**	82.2%	6.9%	31.9%	NC-Unspecified diagnosis (30.3%)	NC-Neurologic (15.9%)	C-Unspecified infection (15.7%)
**Immunologic (156)**	60.8%	8.1%	51.7%	C-Unspecified infection (32.9%)	NC-Unspecified diagnosis (23.2%)	NC-Intoxication/Injury (8.1%)
- **FVs (22)**	55.3%	0.8%	67.1%	C-Unspecified infection (40.5%)	NC-Unspecified diagnosis (14.6%)	C-Respirator (10.3%)

**Craniofacial (162)**	53.0%	21.5%	41.2%	NC-Intoxication/injury (21.5%)	C-Unspecified infection (21.5%)	NC-Congenital malformation (14.1%)
- **FVs (21)**	76.2%	2.6%	41.2%	NC-Unspecified diagnosis (31.2%)	C-Unspecified infection (19.4%)	NC- Congenital malformation (9.8%)
**Metabolic (124)**	65.2%	9.3%	39.9%	NC-Unspecified diagnosis (27.7%)	C-Unspecified infection (21.1%)	NC-Neurologic (12.3%)
- **FVs (20)**	63.0%	5.2%	44.0%	NC-Unspecified diagnosis (32.7%)	C-Gastrointestinal infection (18.9%)	C-Unspecified infection (15.2%)
**Urogenital (98)**	42.1%	23.1%	46.0%	**C-Urinary infection (21.5%)**	**NC-Urogenital (17.4%)**	C-Unspecified infection (13.6%)
- **FVs (10)**	55.1%	5.1%	57.9%	C-Unspecified infection (34.4%)	NC-Unspecified diagnosis (27.0%)	**C-Urinary infection (14.4%)**
**Endocrinologic (67)**	77.7%	8.4%	30.8%	**NC-Endocrinologic (32.4%)**	NC-Unspecified diagnosis (14.9%)	C-Unspecified infection (11.2%)
- **FVs (6)**	78.5%	10.2%	23.4%	NC-Unspecified diagnosis (17.7%)	NC-Neurologic (16.9%)	**NC-Endocrinologic (15.0%)**
**Psychological (72)**	100.7%	8.4%	29.7%	**NC-Psychological (56.1%)**	NC-Intoxication/Injury (29.7%)	NC-Neurologic (6.1%)
- **FVs (5)**	79.4%	16.2%	10.7%	**NC-Psychological (57.4%)**	NC-Intoxication/Injury (16.2%)	NC-Gastrointestinal (6.2%)
**Dermatologic (3)**	-	-	-	-	-	-
**No comorbidity (7,066)**	38.8%	48.5%	23.4%	NC-Intoxication/Injury (48.5%)	NC-Unspecified diagnosis (16.1%)	C-Unspecified infection (11.4%)
- **FVs (111)**	56.0%	11.0%	44.0%	C-Unspecified infection (21.4%)	NC-Unspecified diagnosis (20.1%)	NC-Gastrointestinal (12.4%)

**Because children could have more than one diagnoses category rows do not add up to 100%.*
Diagnoses corresponding to the body system of the comorbidity are marked bold.
NC: Non-Communicable; I: Intoxication/injuries; C: Communicable.
